# Association of Internet Addiction With Nonsuicidal Self-injury Among Adolescents in China

**DOI:** 10.1001/jamanetworkopen.2020.6863

**Published:** 2020-06-04

**Authors:** Jie Tang, Ying Ma, Stephen P. Lewis, Ruoling Chen, Angela Clifford, Brooke A. Ammerman, Marufu Martin Gazimbi, Adrian Byrne, Yu Wu, Xinchuan Lu, Hongjuan Chang, Chun Kang, Henning Tiemeier, Yizhen Yu

**Affiliations:** 1 Department of Preventive Medicine, School of Public Health, Guangzhou Medical University, Xinzao, Panyu District, Guangzhou, P. R. China; 2 Faculty of Education, Health and Wellbeing, University of Wolverhampton, Wolverhampton, United Kingdom; 3 Department of Child Healthcare, Guangzhou Women and Children’s Medical Center, Guangzhou Medical University, Guangzhou, P. R. China; 4 Department of Psychology, University of Guelph, Guelph, Ontario, Canada; 5 Department of Psychology, University of Notre Dame, Notre Dame, Indiana; 6 Global Development Institute, ALB, University of Manchester, Manchester, United Kingdom; 7 School of Medicine, Queen’s Medical Centre, University of Nottingham, Nottingham, United Kingdom; 8 Shenzhen Center for Disease Control and Prevention, Nanshan District, Shenzhen, P. R. China; 9 Department of Child and Adolescent Psychiatry, Suzhou Guangji Hospital, Suzhou, P. R. China; 10 Department of Maternal and Child Healthcare, School of Public Health, Tongji Medical College, Huazhong University of Science and Technology, Hankou District, Wuhan, P. R. China; 11 Department of Child and Adolescent Psychiatry, Erasmus University Medical Centre–Sophia Children's Hospital, Rotterdam, the Netherlands; 12 Department of Social and Behavioral Sciences, Harvard T. H. Chan School of Public Health, Boston, Massachusetts

## Abstract

**Importance:**

Both nonsuicidal self-injury (NSSI), defined as the direct, deliberate damage of one's body tissue without suicidal intent, and internet addiction among adolescents are public health concerns. However, the possible association of NSSI with internet addiction is not well understood.

**Objective:**

To examine the occurrence of internet addiction with NSSI and any sex differences among Chinese adolescents.

**Design, Setting, and Participants:**

A multicenter, cross-sectional, survey study was conducted from February 18 to October 15, 2015, among adolescents aged 11 to 20 years from 343 classes in 45 public high schools across 5 provinces of China. Data analysis was performed from August 1, 2018, to March 1, 2019.

**Exposures:**

Possible internet addiction and internet addiction.

**Main Outcomes and Measures:**

Less-frequent (1-4 times) NSSI and more-frequent (≥5 times) NSSI were surveyed using the Chinese version of the Functional Assessment of Self-Mutilation.

**Results:**

A total of 15 623 students (8043 male [51.5%] and 7580 female [48.5%]) aged 11 to 20 years (mean [SD] age, 15.1 [1.8] years) participated. Of these, 4670 participants (29.9%) met the criteria for possible internet addiction and 509 participants (3.3%) met the criteria for internet addiction. A total of 2667 students (17.1%) engaged in less-frequent NSSI, while 1798 students (11.5%) engaged in more-frequent NSSI in the 12 months preceding the survey. Both possible internet addiction and internet addiction were associated with less-frequent or more-frequent NSSI. The adjusted odds ratios were 1.29 (95% CI, 1.17-1.42) for possible internet addiction and 1.41 (95% CI, 1.11-1.80) for internet addiction for less-frequent NSSI; for more-frequent NSSI, the adjusted odds ratios were 1.75 (95% CI, 1.56-1.96) for possible internet addiction and 2.66 (95% CI, 2.10-3.38) for internet addiction. These associations were similarly observed among age groups of 11 to 14, 15 to 17, and 18 to 20 years. No sex disparities were found in the associations of internet addiction with NSSI, except among adolescents aged 11 to 14 years, where the odds ratios for possible internet addiction with less-frequent NSSI were higher in male adolescents (1.53; 95% CI, 1.25-1.88) than female adolescents (1.13; 95% CI, 0.90-1.47).

**Conclusion and Relevance:**

Internet addiction appears to be associated with NSSI, and the findings of this study suggest that the association was similar between male adolescents and female adolescents. These data suggest that evaluation of the risk of NSSI for adolescents in association with internet addiction may help health care professionals in developing preventive interventions for NSSI.

## Key Points

**Question:**

Is internet addiction associated with nonsuicidal self-injury, and, if so, are there sex differences in the association?

**Findings:**

In this survey study of 15 623 adolescents in China, both possible internet addiction and internet addiction were associated with nonsuicidal self-injury. There were no differences in the associations by sex or age.

**Meaning:**

Interventions to address nonsuicidal self-injury should acknowledge the apparent association with internet addiction.

## Teaser

This survey study examines the possible association between internet addiction and nonsuicidal self-injury in adolescents in China.

## Introduction

Nonsuicidal self-injury (NSSI), defined as the direct, deliberate damage of one's body tissue (eg, cutting, burning) without suicidal intent, is widespread among adolescents worldwide.^1^ The prevalence rates of NSSI can be as high as 1 in 5 in community settings, with higher prevalence rates reported in clinical samples of youths.^2^ Engagement in NSSI is associated with various mental difficulties, including, but not limited to depression, anorexia nervosa,^3^ substance use,^4^ attention-deficit/hyperactivity disorder, and oppositional defiant disorder.^5^ Moreover, evidence suggests that NSSI is a unique and notable risk factor for suicidal thoughts and behaviors.^6^ In this way, NSSI represents a public health concern regarding adolescents.

One area gaining increasing attention in the field is the possible link between NSSI and internet addiction among youth. Internet addiction, characterized by excessive or poorly controlled preoccupations, urges, or behaviors regarding internet access, has also emerged as a concern among adolescents given its numerous adverse outcomes with physical and emotional manifestations, such as depression, anxiety, and loneliness.^7,8^ To date, several studies have examined the association of internet addiction with self-harm,^2,9,10,11,12,13,14^ a broader term that includes NSSI but also encompasses suicidal behaviors. However, findings from these studies have been inconsistent. Previous studies indicated that both NSSI and internet addiction have functions of emotional regulation^15,16^; however, to our knowledge, no study that examined the association of NSSI with internet addiction has adjusted for confounders related to emotional regulation, such as emotional management ability, psychological resilience, and loneliness. Taken together, this lack of information makes it difficult to fully understand the extent to which internet addiction and NSSI are associated. In addition, previous studies have reported that the epidemiologic characteristics of both internet addiction and NSSI may have sex differences,^3,11^ yet few studies have examined whether there are sex differences in the association of internet addiction with NSSI. Owing to the number of adverse consequences of both internet addiction and NSSI, addressing limitations in previous research is essential to not just gain a fuller understanding of how internet addiction and NSSI interrelate, but also for health professionals monitoring risk factors among adolescents with NSSI and/or internet addiction, and for public health efforts to identify those at risk for NSSI and/or internet addiction.

The present study aimed to investigate the possible association of internet addiction with NSSI specifically and understand whether sex differences are present within the context of this association. We hypothesized that internet addiction would be associated with NSSI and that the association would differ between male and female adolescents, even after adjustment for various confounders.

## Methods

### Study Participants and Data Collection

We conducted a cross-sectional survey among adolescents in grades 7 to 12 across 5 representative provinces (Heilongjiang [Northern]; Anhui [Eastern]; Guangdong [Southern]; Yunnan [Western]; and Hubei [Central]) in China, from February 18 to October 15, 2015. The survey aimed to note the epidemiologic characteristics of abnormal behaviors (mainly aggressive behavior and NSSI) among Chinese adolescents and provide evidence for forming prevention and therapy interventions. In each province, we used the same sampling method to recruit participants and the same questionnaires to collect data. The survey-related design, organization, and implementation have been described.^17^ Briefly, we used a multistage, random cluster sampling method to select students. First, with the help of local educational bureaus, we selected 9 high schools in each province. In each selected school, we then used random digits to choose 2 to 3 classes from each grade (grades 7-9 in junior high schools and grades 10-12 in senior high schools). All students in the selected classes were eligible to participate in the survey except for those with severe mental disorders (eg, severe depression, schizophrenia, paranoid psychosis, and bipolar disorder) who were identified by the head teacher and/or the health care physicians. Consent forms were sent to a total of 15 797 students of 343 classes in 45 public high schools (27 junior high schools and 18 senior high schools) by the head teacher of each selected class to ask for their participation. All of the interested students or their guardians (if the student was younger than 14 years) provided written informed consent before participation in the survey. Seventy-eight students refused to participate in the survey, 21 students were absent from school on the day of the survey, and 75 students submitted an incomplete questionnaire with missing data for more than 15% of the items on the questionnaire. Therefore, the remaining 15 623 participants were included in the current analysis; the response rate was 98.9%. Participants did not receive financial compensation. The questionnaires were anonymous. This study received ethics clearance from Guangzhou Medical University and Huazhong University of Science and Technology. This study followed the American Association for Public Opinion Research (AAPOR) reporting guideline.

Trained investigators (teachers and postgraduates) conducted the survey and they were available at each site to clarify the student’s possible confusion and questions about the structured questionnaire. Participants completed the survey in a single sitting while at school. Before the survey, all participants were informed of the purposes and procedures of the study in detail. All participants were required to complete the anonymous questionnaire independently within 30 to 35 minutes. Completeness of questionnaires was reviewed by investigators before the participants left the site.

### Instruments

Participants’ possible NSSI was measured by using the Chinese version of the Functional Assessment of Self-Mutilation, which allowed for an assessment of the methods, frequencies, and purposes of NSSI over the past 12 months.^18^ In particular, participants were asked to report how frequently they had engaged in 8 different forms of NSSI (ie, hitting, head banging, stabbing, pinching, scratching, biting, burning, and cutting) in the past year. To distinguish between NSSI and suicidal behaviors, participants were also asked whether any of those behaviors carried suicide intent. For the purpose of this study and similar to previous research,^17,18^ participants were divided into 3 categories based on the frequency of NSSI: (1) engaged in NSSI 5 or more times, defined as more-frequent NSSI^19^; (2) engaged in NSSI 1 to 4 times, defined as less-frequent NSSI^17,20^; and (3) did not engage in NSSI, defined as non-NSSI. The Chinese version of the Functional Assessment of Self-Mutilation has demonstrated satisfactory psychometric properties, yielding an acceptable internal consistency with a Cronbach α range from 0.76 to 0.81.^21^

Internet addiction was measured by using the Young Internet Addiction Test (IAT), which is widely used when assessing internet addiction (eTable 1 in the Supplement).^22^ The IAT comprises 20 summed items using a 5-point Likert scale: 1, rarely; 2, occasionally; 3, frequently; 4, often; and 5, always; thus, total scores on the IAT range from 20 to 100. Three types of internet user groups were identified based on the original cutoff points proposed by Young.^23^ Respondents with IAT scores between 70 and 100 were classified as addicted and most likely had encountered significant life problems due to excessive internet use. Respondents with IAT scores between 40 and 69 were classified as possibly addicted (possible IA). Respondents with IAT scores between 20 and 39 were classified as nonaddicted (non–internet addiction) and most likely to be average internet users.^24^ The IAT has been demonstrated to have acceptable internal consistency, with a Cronbach α coefficient of 0.93 in a previous study^24^ and 0.93 in the present study sample.

Psychological resilience was measured using the Resilience Scale for Chinese Adolescents (eTable 2 in the Supplement).^51^ It has 27 items using a 5-point Likert scale (1, totally inconsistent; 2, inconsistent; 3, not sure; 4, consistent; and 5, fully consistent), with higher total scores indicating better psychological resilience. Previous studies have shown that the Resilience Scale for Chinese Adolescent has good reliability and validity, and the Cronbach α coefficient of the scale was 0.90.^25^ In the present study sample, the Cronbach α coefficient was 0.76.

A 4-item subscale from the Emotional Intelligence Inventory was used to measure emotional management ability (eTable 3 in the Supplement),^26^ with 4-point Likert scale responses: 1, always like this; 2, often like this; 3, rarely like this; and 4, never like this. Higher total scores represent greater emotional management ability. The scale was demonstrated as having an acceptable internal consistency in a previous study (Cronbach α coefficient = 0.83)^17^ and in the present study (Cronbach α coefficient = 0.77).

Loneliness was measured by using the revised version of the Loneliness Scale, which was developed by Li et al^27^ (eTable 4 in the Supplement). The Loneliness Scale has 21 items with 5-point Likert scale responses (1, totally inconsistent; 2, inconsistent; 3, not sure; 4, consistent; and 5, fully consistent). Higher total scores indicate greater loneliness. The scale has been demonstrated as having acceptable internal consistency; the Cronbach α coefficient value was 0.76 and 0.85 in the previous study^27^ and 0.78 in the present study.

Suicidal ideation was measured by the question, “During the past 12 months, did you ever seriously consider attempting suicide?” Suicidal ideation was defined as an affirmative answer to this question. Suicide attempt was measured by the question, “During the past 12 months, how many times did you actually attempt suicide?” Suicide attempt was defined as once or more. A previous study indicated that these questions have good reliability and validity.^28^

Previous studies have suggested that both NSSI and internet addiction were associated with a wide range of demographic characteristics and socioeconomic status,^15,16^ which should be adjusted when examining the association of internet addiction with NSSI. We used a self-designed questionnaire to collect socioeconomic, familial, and parenting variables, including age, sex, ethnicity, family income, family structure, single-child family, parents' educational level, and parenting style. A previous study had shown that the test-retest reliability of the questionnaires was α = 0.83.^29^

### Statistical Analysis

Data analysis was performed from August 1, 2018, to March 1, 2019. Frequencies and proportions for categorical variables or mean (SD) for continuous variables were used to describe the characteristics of the participants and less-frequent or more-frequent NSSI among the participants by different characteristics. In addition, χ^2^ tests or 2-tailed, unpaired *t* tests were used to compare the distribution between male and female participants according to different characteristics.

To aid in understanding the association of internet addiction and NSSI, multinominal logistic regression analyses were used to estimate the adjusted odds ratios (aORs) and 95% CIs of internet addiction and possible internet addiction for participants engaged in less-frequent or more-frequent NSSI separately, and different covariates were adjusted in 3 models to examine the robustness of the associations. In model 1, we adjusted for demographic characteristics of participants, including provinces (Anhui, Guangdong, Heilongjiang, Hubei, and Yunnan), sex (male or female), age groups (11-14, 15-17, and 18-20 years), grade (junior high school or senior high school), and ethnicity (Han or others). In model 2, we also adjusted for covariates of socioeconomic status and family environment, including family income (<$150, $150-850, or >$850/mo), which was divided according to local economic level and referred to in a previous study,^30^ family structure (single parent/restructured family, grandparents’ family, combined family, or core family/stem family), single-child family (yes or no), parents’ educational level (college or above, senior high school/technical school, or junior high school or below), parenting styles (strict, pampered, neglect or frequently changing, or open-minded). In model 3, we additionally adjusted for covariates of psychology and comorbidities, including emotional management ability (continuous data), psychological resilience (continuous data), loneliness (continuous data), suicidal ideation (yes or no), and suicide attempt (yes or no).

We conducted subgroup analysis to examine whether the associations of internet addiction with NSSI were different by age or sex, and any sex differences in the associations were examined via calculating a ratio of ORs.^31^ Sex differences were also analyzed by age.

We assigned missing data to a separate category in the covariates.^32^ Significance level was set at *P* < .05 and all tests were 2-sided. Statistical analyses were conducted using SPSS Statistics, version 25.0 (IBM Corp).

## Results

We included 15 623 adolescents with 8043 male participants (51.5%) and 7580 female participants (48.5%) in the final analyses. The sample came from Anhui Province (21.1%), Guangdong Province (19.7%), Heilongjiang Province (18.5%), Hubei Province (19.3%), and Yunnan Province (21.4%). The age of the participants ranged from 11 to 20 years, with most (60.1%) aged between 15 and 20 years; the mean (SD) age was 15.1 (1.8) years. More than half (53.4%) of the participants were junior high school students, 90.7% were Han nationality, 34.6% were from single-child families, and 87.2% were from a core family/stem family. Other characteristics are reported in Table 1.

**Table 1.** Characteristics of Participants

Variable	No. (%)				
	Sex		Total (n = 15 623)	NSSI	
	Male (n = 8043)	Female (n = 7580)		Less frequent (n = 2667)	More frequent (n = 1798)
Province^a^					
Anhui	1856 (23.1)	1446 (19.1)	3302 (21.1)	655 (24.6)	395 (22.0)
Guangdong	1530 (19.0)	1544 (20.4)	3074 (19.7)	603 (22.6)	378 (21.0)
Heilongjiang	1411 (17.5)	1478 (19.5)	2889 (18.5)	351 (13.2)	164 (9.1)
Hubei	1640 (20.4)	1372 (18.1)	3012 (19.3)	558 (20.9)	342 (19.0)
Yunnan	1606 (20.0)	1740 (23.0)	3346 (21.4)	500 (18.7)	519 (28.9)
Age, y					
11-14	3202 (39.8)	3026 (39.9)	6228 (39.9)	1115 (41.8)	767 (42.7)
15-17	2770 (34.4)	2551 (33.7)	5321 (34.1)	878 (32.9)	611 (34.0)
18-20	2071 (25.7)	2003 (26.4)	4074 (26.1)	674 (25.3)	420 (23.4)
Grade					
Junior high	4312 (53.6)	4024 (53.1)	8336 (53.4)	1404 (52.6)	1007 (56.0)
Senior high	3731 (46.4)	3556 (46.9)	7287 (46.6)	1263 (47.4)	791 (44.0)
Ethnicity^a^					
Han	7406 (92.1)	6757 (89.1)	14 163 (90.7)	2460 (92.2)	1538 (85.5)
Other	637 (7.9)	823 (10.9)	1460 (9.3)	207 (7.8)	260 (14.5)
Single-child family^a^					
Yes	3374 (42.0)	2037 (26.9)	5411 (34.6)	854 (32.0)	568 (31.6)
No	4669 (58.1)	5543 (73.1)	10 212 (65.4)	1813 (68.0)	1230 (68.4)
Family structure^a^					
Single parent/recombine family	534 (6.6)	538 (7.1)	1072 (6.9)	174 (6.5)	160 (8.9)
Grandparents	243 (3.0)	235 (3.1)	478 (3.1)	89 (3.3)	74 (4.1)
Combined family	204 (2.5)	253 (3.3)	457 (2.9)	95 (3.6)	77 (4.3)
Core family/stem family	7062 (87.8)	6554 (86.5)	13 616 (87.2)	2309 (86.6)	1487 (82.7)
Family income, $/mo^a^					
<150	1278 (15.9)	1430 (18.9)	2708 (17.3)	453 (17.0)	341 (19.0)
150-850	5579 (69.4)	5263 (69.4)	10 842 (69.4)	1876 (70.3)	1202 (66.9)
>850	1186 (14.8)	887 (11.7)	2073 (13.3)	338 (12.7)	255 (14.2)
Father's educational level					
College or above	695 (8.6)	622 (8.2)	1317 (8.4)	228 (8.5)	196 (10.9)
Senior high school/technical school	1488 (18.5)	1393 (18.4)	2881 (18.4)	484 (18.1)	351 (19.5)
Junior high school or below	5860 (72.9)	5565 (73.4)	11 425 (73.1)	1955 (73.3)	1251 (69.6)
Mother's educational level					
College or above	484 (6.0)	433 (5.7)	917 (5.9)	135 (5.1)	136 (7.6)
Senior high school/technical school	1064 (13.2)	1093 (14.4)	2157 (13.8)	370 (13.9)	265 (14.7)
Junior high school or below	6495 (80.8)	6054 (79.9)	12 549 (80.3)	2162 (81.0)	1397 (77.7)
Parenting style^a^					
Strict	2459 (30.6)	2261 (29.8)	4720 (30.2)	774 (29.0)	548 (30.5)
Pamper	352 (4.4)	207 (2.7)	559 (3.6)	103 (3.9)	74 (4.1)
Neglect/frequently changing	954 (11.9)	728 (9.6)	1682 (10.8)	358 (13.4)	284 (15.8)
Open-minded	3913 (48.7)	4102 (54.1)	8015 (51.3)	1329 (49.8)	831 (46.2)
Missing data	365 (4.5)	282 (3.7)	647 (4.1)	103 (3.9)	61 (3.4)
Emotion management, mean (SD)^a^	11.7 (2.9)	11.3 (2.7)	11.5 (2.8)	10.9 (2.6)	10.3 (3.0)
Psychological resilience, mean (SD)^a^	91.2 (13.1)	93.0 (12.9)	92.1 (13.1)	90.3 (12.3)	87.7 (12.4)
Loneliness, mean (SD)^a^	50.5 (10.4)	49.23 (10.0)	49.9 (10.2)	51.6 (10.1)	54.6 (11.0)
Suicidal ideation^a^					
Yes	1114 (13.9)	1243 (16.4)	2357 (15.1)	664 (24.9)	759 (42.2)
No	6857 (85.3)	6276 (82.8)	13 133 (84.1)	1989 (74.6)	1033 (57.5)
Missing data	72 (0.9)	61 (0.8)	133 (0.9)	14 (0.5)	6 (0.3)
Suicide attempt					
Yes	260 (3.2)	279 (3.7)	539 (3.5)	146 (5.5)	248 (13.8)
No	7700 (95.7)	7231 (95.4)	14 931 (95.6)	2508 (94.0)	1543 (85.8)
Missing data	83 (1.0)	70 (0.9)	153 (1.0)	13 (0.1)	7 (0.4)
Status of internet addiction^a^					
Non–internet addiction	4772 (59.3)	5672 (74.8)	10 444 (66.9)	1622 (60.8)	867 (48.2)
Possible internet addiction	2910 (36.2)	1760 (23.2)	4670 (29.9)	936 (35.1)	783 (43.5)
Internet addiction	361 (4.5)	148 (2.0)	509 (3.3)	109 (4.1)	148 (8.2)

Abbreviation: NSSI, nonsuicidal self-injury.

^a^ Distributions of sex with respect to different characteristics were statistically significant (*P* < .05).

Overall, 4670 participants met the criteria of possible internet addiction and 509 participants met the criteria of internet addiction. The prevalence rates of possible internet addiction and internet addiction were 29.9% and 3.3%, respectively, with male participants showing a higher prevalence rate of possible internet addiction and internet addiction. There were 2667 participants (17.1%) who engaged in NSSI 1 to 4 times during the past 12 months before the survey (ie, less frequent). There were 1798 participants (11.5%) who engaged in NSSI 5 or more times during the past 12 months before the survey (ie, more frequent). Female particpants had a significantly higher prevalence rate of less-frequent NSSI than male participants (eTable 5 in the Supplement). Participants who had possible internet addiction or internet addiction had poorer emotional regulation ability (*F* = 503.14, *P* < .001) and more loneliness (*F* = 554.82, *P* < .001) (eTable 6 in the Supplement).

The prevalence rate of less-frequent NSSI among participants with non–internet addiction was 15.5%; possible internet addiction, 20.0%; and internet addiction, 21.4%. The prevalence rate of more-frequent NSSI among participants with non–internet addiction was 8.3%; possible internet addiction, 16.8%; and internet addiction, 29.1%. Possible internet addiction and internet addiction were positively associated with less-frequent or more-frequent NSSI. The unadjusted ORs and aORs are presented in Table 2. The aORs did not change substantially from model 1 to model 2. However, following adjustment for psychological factors and comorbidities in model 3, the aORs were reduced substantially. In the fully adjusted model (model 3) for less-frequent NSSI, the aOR for possible internet addiction was 1.29 (95% CI, 1.17-1.42) and, for internet addiction, 1.41 (95% CI, 1.11-1.80). For more-frequent NSSI, the aOR for possible internet addiction was 1.75 (95% CI, 1.56-1.96) and, for internet addiction, 2.66 (95% CI, 2.10-3.38). Significant associations of possible internet addiction and internet addiction with less-frequent or more-frequent NSSI were also found among different age groups of 11 to 14 years, 15 to 17 years, and 18 to 20 years, except for the null associations of internet addiction with less-frequent NSSI in age groups of 11 to 14 years and 18 to 20 years (Table 2). For example, for less-frequent NSSI among participants aged 15 to 17 years, the aOR for possible internet addiction was 1.27 (95% CI, 1.07-1.50), and for internet addiction, 1.45 (95% CI, 1.06-1.84). For more-frequent NSSI among participants aged 15 to 17 years, the aOR for possible internet addiction was 1.63 (95% CI, 1.34-1.98), and for internet addiction, 1.88 (95% CI, 1.26-2.82).

**Table 2.** Less-Frequent and More-Frequent NSSI by Possible Internet Addiction and Internet Addiction in Age Groups

Age group	No. (%)	OR (95% CI)			
		Unadjusted	Model 1^a^	Model 2^b^	Model 3^c^
**Total**					
Less-frequent NSSI					
Non–internet addiction	1622 (15.5)	1 [Reference]	1 [Reference]	1 [Reference]	1 [Reference]
Possible internet addiction	936 (20.0)	1.56 (1.42-1.70)	1.63 (1.49-1.79)	1.61 (1.47-1.77)	1.29 (1.17-1.42)
Internet addiction	109 (21.4)	2.12 (1.68-2.67)	2.32 (1.84-2.94)	2.20 (1.74-2.78)	1.41 (1.11-1.80)
More-frequent NSSI					
Non–internet addiction	867 (8.3)	1 [Reference]	1 [Reference]	1 [Reference]	1 [Reference]
Possible internet addiction	783 (16.8)	2.44 (2.19-2.71)	2.55 (2.29-2.84)	2.52 (2.25-2.81)	1.75 (1.56-1.96)
Internet addiction	148 (29.1)	5.39 (4.35-6.68)	6.12 (4.91-7.64)	5.64 (4.51-7.05)	2.66 (2.10-3.38)
**11-14 y**					
Less-frequent NSSI					
Non–internet addiction	725 (16.5)	1 [Reference]	1 [Reference]	1 [Reference]	1 [Reference]
Possible internet addiction	353 (21.7)	1.66 (1.43-1.92)	1.74 (1.50-2.02)	1.69 (1.46-1.97)	1.33 (1.14-1.56)
Internet addiction	37 (19.0)	2.05 (1.38-3.05)	2.30 (1.54-3.43)	2.15 (1.44-3.21)	1.46 (0.94-2.01)
More-frequent NSSI					
Non–internet addiction	384 (8.7)	1 [Reference]	1 [Reference]	1 [Reference]	1 [Reference]
Possible internet addiction	307 (18.9)	2.72 (2.31-3.22)	2.85 (2.40-3.39)	2.81 (2.36-3.35)	1.96 (1.64-2.36)
Internet addiction	76 (39.0)	7.96 (5.72-11.06)	9.36 (6.65-13.17)	8.81 (6.23-12.43)	4.17 (2.89-6.01)
**15-17 y**					
Less-frequent NSSI					
Non–internet addiction	498 (14.8)	1 [Reference]	1 [Reference]	1 [Reference]	1 [Reference]
Possible internet addiction	335 (19.0)	1.52 (1.31-1.78)	1.59 (1.35-1.86)	1.57 (1.34-1.85)	1.27 (1.07-1.50)
Internet addiction	45 (22.2)	2.08 (1.45-2.98)	2.31 (1.61-3.34)	2.18 (1.51-3.15)	1.45 (1.06-1.84)
More-frequent NSSI					
Non–internet addiction	280 (8.3)	1 [Reference]	1 [Reference]	1 [Reference]	1 [Reference]
Possible internet addiction	285 (16.2)	2.31 (1.93-2.76)	2.34 (1.95-2.81)	2.29 (1.90-2.76)	1.63 (1.34-1.98)
Internet addiction	46 (22.7)	3.79 (2.63-5.45)	4.16 (2.86-6.03)	3.73 (2.56-5.46)	1.88 (1.26-2.82)
**18-20 y**					
Less-frequent NSSI					
Non–internet addiction	399 (14.9)	1 [Reference]	1 [Reference]	1 [Reference]	1 [Reference]
Possible internet addiction	248 (19.3)	1.53 (1.28-1.83)	1.56 (1.30-1.87)	1.57 (1.31-1.89)	1.29 (1.07-1.57)
Internet addiction	27 (24.3)	2.42 (1.52-3.88)	2.49 (1.54-4.02)	2.38 (1.47-3.87)	1.49 (0.98-2.44)
More-frequent NSSI					
Non–internet addiction	203 (7.6)	1 [Reference]	1 [Reference]	1 [Reference]	1 [Reference]
Possible internet addiction	191 (14.9)	2.32 (1.87-2.87)	2.34 (1.88-2.92)	2.39 (1.91-2.98)	1.64 (1.29-2.05)
Internet addiction	26 (23.4)	4.59 (2.83-7.45)	4.88 (2.97-8.03)	4.56 (2.74-7.56)	2.01 (1.17-3.44)

Abbreviations: NSSI, nonsuicidal self-injury; OR, odds ratio.

^a^ Adjusted for sex, province, age (in total model), grade, and ethnicity.

^b^ Additionally adjusted for family income, family structure, single-child family, parents’ educational level, and parenting styles.

^c^ Adjusted for emotion management, loneliness, psychological resilience, suicidal ideation, and suicide attempts in addition to the covariates in model 2.

Stratified analyses were also conducted to examine the association of possible internet addiction and internet addiction with less-frequent or more-frequent NSSI in male and female participants (Table 3). Significant associations of internet-addiction with NSSI were found among both male and female participants. However, no significant differences between the sexes were found in the association of possible internet addiction and internet addiction with less-frequent or more-frequent NSSI. Sex differences also were not found in different age groups, except for adolescents aged 11 to 14 years, in which the aORs of possible internet addiction with less-frequent NSSI were significantly higher in male participants (1.53; 95% CI, 1.25-1.88) than female participants (1.13; 95% CI, 0.90-1.47); the ratio of the aORs in male participants vs female participants was 1.35 (*P* = .03).

**Table 3.** Less-Frequent and More-Frequent NSSI by Possible Internet Addiction and Internet Addiction in Male vs Female Participants

Age groups	Male			Female			ROR^c^	*P* value^d^
	No. (%)	OR (95% CI)^a^	OR (95% CI)^b^	No. (%)	OR (95% CI)^a^	OR (95% CI)^b^		
Total								
Less-frequent NSSI								
Non–internet addiction	659 (13.8)	1 [Reference]	1 [Reference]	963 (17.0)	1 [Reference]	1 [Reference]		
Possible internet addiction	557 (19.1)	1.64 (1.45-1.86)	1.44 (1.07-1.93)	379 (21.5)	1.61 (1.41-1.85)	1.15 (1.02-1.35)	1.25	.41
Internet addiction	72 (19.9)	2.05 (1.54-2.71)	1.42 (1.24-1.62)	37 (25.0)	3.07 (2.00-4.69)	1.60 (1.04-2.53)	0.89	.34
More-frequent NSSI								
Non–internet addiction	386 (8.1)	1 [Reference]	1 [Reference]	481 (8.5)	1 [Reference]	1 [Reference]		
Possible internet addiction	433 (14.9)	2.18 (1.88-2.52)	1.64 (1.40-1.92)	350 (19.9)	2.98 (2.56-3.48)	1.83 (1.55-2.16)	0.90	.18
Internet addiction	90 (24.9)	4.37 (3.33-5.72)	2.38 (1.77-3.19)	58 (39.2)	9.62 (6.55-14.13)	3.42 (2.23-5.24)	0.70	.11
11-14 y								
Less-frequent NSSI								
Non–internet addiction	298 (14.7)	1 [Reference]	1 [Reference]	427 (18.0)	1 [Reference]	1 [Reference]		
Possible internet addiction	225 (21.6)	1.80 (1.48-2.19)	1.53 (1.25-1.88)	128 (21.9)	1.66 (1.32-2.09)	1.13 (0.90-1.47)	1.35	.03
Internet addiction	24 (18.3)	1.97 (1.21-3.20)	1.44 (0.89-2.40)	13 (20.3)	2.93 (1.43-6.03)	1.57 (0.75-3.36)	0.92	.43
More-frequent NSSI								
Non–internet addiction	166 (8.2)	1 [Reference]	1 [Reference]	218 (9.2)	1 [Reference]	1 [Reference]		
Possible internet addiction	164 (15.7)	2.36 (1.87-2.98)	1.79 (1.39-2.29)	143 (24.5)	3.63 (2.85-4.63)	2.11 (1.62-2.76)	0.85	.19
Internet addiction	43 (32.8)	6.33 (4.16-9.61)	3.69 (2.34-5.82)	33 (51.6)	14.57 (8.07-26.33)	5.19 (2.68-10.32)	0.71	.24
15-17 y								
Less-frequent NSSI								
Non–internet addiction	200 (12.7)	1 [Reference]	1 [Reference]	298 (16.7)	1 [Reference]	1 [Reference]		
Possible internet addiction	187 (17.8)	1.64 (1.32-2.04)	1.41 (1.11-1.78)	148 (20.8)	1.55 (1.24-1.94)	1.16 (0.92-1.48)	1.22	.13
Internet addiction	31 (20.7)	2.17 (1.40-3.35)	1.49 (0.94-2.38)	14 (26.4)	2.73 (1.39-5.37)	1.65 (0.85-3.30)	0.9	.41
More-frequent NSSI								
Non–internet addiction	127 (8.1)	1 [Reference]	1 [Reference]	153 (8.6)	1 [Reference]	1 [Reference]		
Possible internet addiction	151 (14.4)	2.09 (1.62-2.69)	1.48 (1.12-1.94)	134 (18.8)	2.72 (2.11-3.52)	1.79 (1.35-2.37)	0.83	.18
Internet addiction	30 (20.0)	3.30 (2.10-5.19)	1.60 (1.01-2.67)	16 (30.2)	6.07 (3.14-11.75)	2.82 (1.37-5.83)	0.57	.16
18-20 y								
Less-frequent NSSI								
Non–internet addiction	161 (13.7)	1 [Reference]	1 [Reference]	238 (15.8)	1 [Reference]	1 [Reference]		
Possible internet addiction	145 (17.7)	1.49 (1.16-1.91)	1.34 (1.04-1.75)	103 (22.2)	1.74 (1.33-2.26)	1.23 (0.93-1.65)	1.09	.34
Internet addiction	17 (21.2)	2.11 (1.19-3.77)	1.43 (0.80-2.68)	10 (32.3)	4.06 (1.73-9.50)	1.78 (0.74-4.45)	0.80	.37
More-frequent NSSI								
Non–internet addiction	93 (7.9)	1 [Reference]	1 [Reference]	110 (7.3)	1 [Reference]	1 [Reference]		
Possible internet addiction	118 (14.4)	2.10 (1.57-2.81)	1.66 (1.21-2.27)	73 (15.7)	2.66 (1.73-3.68)	1.57 (1.11-2.23)	1.06	.41
Internet addiction	17 (21.2)	3.65 (2.01-6.63)	2.03 (1.06-3.90)	9 (29.0)	7.90 (3.26-19.17)	2.29 (0.87-6.24)	0.89	.43

Abbreviations: NSSI, nonsuicidal self-injury; OR, odds ratio; ROR, ratio of ORs.

^a^ Unadjusted model.

^b^ Adjusted for province, age (in total model), grade, ethnicity, family income, family structure, single-child family, parents’ educational level, parenting style, emotional management ability, loneliness, psychological resilience, suicidal ideation, and suicide attempt.

^c^ Calculated by adjusted OR.

^d^ One-sided *P* value.

## Discussion

A multicenter cross-sectional survey with a high response rate was conducted to investigate the possible association of internet addiction with NSSI as well as potential sex differences therein. The data revealed that possible internet addiction and internet addiction were positively associated with less-frequent NSSI and more-frequent NSSI among adolescents. Similar associations were found in male and female participants as well as across different age groups. No significant sex differences in these associations of internet addiction with NSSI were observed; however, the association of possible internet addiction with less-frequent NSSI was significantly stronger in male participants than female participants among adolescents aged 11 to 14 years.

### Comparison With Other Studies

Seven studies have examined the association of internet addiction with broader self-harm (sometimes but not exclusively pertaining to NSSI). However, these studies have indicated inconsistent results.^2,9,10,11,12,13,14^ Five studies have suggested that internet addiction is associated with self-harm,^2,10,11,12,13^ while 2 other studies suggested a null association of internet addiction with self-harm.^9,14^ The findings in our multicenter survey were consistent with those of most previous studies, which found that internet addiction was associated with NSSI.^2,10,11,12,13^ The inconsistent results among previous studies may be associated with various characteristics within the study population, such as different prevalence of self-harm and internet addiction that was evaluated by different measurements and criteria, apart from study design and adjustments. Studies that do not distinguish between NSSI and self-harm may have overestimated the association of internet addiction with NSSI.^10,11,12^ Thus, the discrepancies of the association between internet addiction and NSSI highlight the need to establish a more-uniform evaluative measurement of NSSI apart from broader self-harm behavior when conducting this line of research.^17^

To our knowledge, only 1 study has investigated sex differences in the association of internet addiction with self-harm.^11^ Kaess and colleagues^11^ conducted a cross-sectional study of 11 356 adolescents in 11 European countries and found no sex difference in the association of problematic internet use with self-harm, which mirrors findings of the present study. Both internet addiction and NSSI have the function of emotional regulation, and previous studies reported that female participants had higher rates of emotional disorders and tended to ruminate, avoid, and be less active in their coping methods, while male participants were more likely to engage in physical and instrumental forms of coping strategy.^33,34^ Given significant sex differences in coping strategies and epidemiologic characteristics of internet addiction and NSSI,^3,6,11^ further investigation of sex differences in the association of internet addiction with NSSI is needed.

A previous study suggested that the typical onset of NSSI occurs at ages 12 to 13 years, peaks at 14 to 15 years, and then may decline, potentially replaced by substance use in some cases after 16 to 17 years.^35^ Other research indicated that NSSI may have a second peak onset at the time individuals begin university studies (late adolescence/early adulthood).^36^ Because the prevalence of NSSI may vary in terms of its course, it is possible that sex differences in the association of internet addiction with NSSI is mediated by age. In the present study, among adolescents aged 11 to 14 years, we also found that male participants with possible internet addiction had a higher prevalence rate of less-frequent NSSI than female participants, which suggests that internet addiction interventions in boys aged 11 to 14 years may have a greater association with NSSI prevention. However, whether this sex difference is mediated by age or is random needs to be ascertained through further research.

### Possible Explanations of the Association

The causes of NSSI are complex and multifactoral.^37^ Therefore, it is difficult to infer whether internet addiction leads to NSSI or vice versa. However, it is conceivable that part of the association between NSSI and internet addiction stems from a shared association with other factors. In particular, adolescents who engaged in NSSI and internet addiction may have more psychological distress and use these behaviors to obtain what they consider to be relief from distress. In other words, adolescents may use internet addiction and/or NSSI to regulate emotion.^38^ For example, Kitazawa and colleagues^39^ suggested that depression and anxiety can predict internet addiction in university students, and Chwaszcz and colleagues^40^ reported a possible association between internet addiction and the use of coping strategies, such as disengagement, substance use, and self-blame. In the present study, we also found that adolescents with possible internet addiction or internet addiction had poorer emotional regulation ability and more loneliness. From this perspective, it may be that internet addiction is indirectly associated with NSSI, and both factors potentially have a similar function, although more inquiry is needed before conclusions can be made.

In addition, a growing body of evidence suggests that many adolescents who engage in self-injury go online, perhaps more than peers who do not self-injure.^41^ Although there are many reasons for the online activities of adolescents with NSSI,^42^ one reason may be that more engagement in online activity becomes problematic and could potentially contribute to development of internet addiction. Conversely, adolescents with internet addiction or high-level internet use may be more likely to come across NSSI-related information given the amount of time they spend online.^43^ For example, previous studies have reported that youth may be exposed to an array of NSSI websites that may have graphic material that has been cited as potentially triggering for NSSI.^44,45,46^ A recent study has also provided possible evidence that exposure to NSSI imagery on Instagram may elevate risk for future NSSI, even for youth who did not initially experience self-injury.^47^ In sum, whether there is unidirectional or bidirectional association of internet addiction with NSSI should be addressed in future studies.

### Strengths and Limitations

One strength of the present study is the representativeness of the sample within China. For this multicenter study, we used a multistage, random cluster sampling method to recruit 15 623 adolescents across 5 provinces, whereby the social economy and cultures reflect the status in China. In addition, extensive and significant confounders adjusted for in our multivariable analysis and our more-focused assessment of NSSI vs broader self-harm may make the association of internet addiction with NSSI more valid and robust than that of previous studies. The inclusion of sex differences in the analysis also provided possibly valuable sex-specific information that may aid in prevention and treatment of both NSSI and internet addiction, although we found no significant sex differences in the association of internet addiction with NSSI among most age groups.

This study has some limitations. First, data were based on a retrospective self-report of the prevalence of NSSI and internet addiction, which may introduce potential problems with underreporting and bias recall. On balance, systematic reviews have demonstrated that information garnered from a young person and school-based students regarding self-harm and risk factors is likely to be reliable, and such data are useful when prospective data are not available.^48^ Second, the age span of the sample range is large, and the younger students may not have the same capacity to fully understand the questionnaire as older students; nonetheless, we used a standard interview procedure across all participants. Third, although we excluded students with severe mental disorders from this study, we did not assess other mental difficulties, such as depression and anorexia nervosa; thus, we cannot adjust for these potential confounders when assessing the association of internet addiction with NSSI, which may have led to spurious and/or underestimation of the strength of these associations. Fourth, although we performed subgroup analysis to possibly support the association between internet addiction and NSSI and their sex differences, several subgroup analyses included a smaller number of adolescents who had engaged in NSSI, which may lead to unstable or biased estimates of the size of the associations of internet addiction with NSSI. Fifth, all of the participants were from public schools in China, which may affect the generalizability of the results, although most high schools are public in China. Sixth, this study used a cross-sectional design, which precludes conclusions regarding any causal associations between internet addiction and NSSI.

## Conclusions

Our findings have potential public health and clinical implications. The results suggest that NSSI is common and may have a serious influence on well-being among adolescents and necessitates monitoring and early intervention. Given the difficulty in recognizing and treating NSSI when working with youths, assessing young people’s internet use and engaging in early intervention of internet addiction among adolescents may provide an opportunity for identification and subsequent intervention of NSSI. Findings from the present study may also help clinicians who work with adolescents who engage in NSSI to ask about the level of internet use and vice versa. In line with published clinical guidelines, incorporating adolescents’ internet use when working with youth who self-injure is important and may help to facilitate treatment.^49,50,51^

In this multicenter investigation conducted in China, possible internet addiction and internet addiction appeared to be positively associated with less-frequent or more-frequent NSSI among adolescents. Hence, evaluating the risk of NSSI among adolescents with internet addiction is necessary. The present findings also underscore the need for additional research. In particular, further investigation is warranted to determine whether there is a causal association and possible sex differences between internet addiction and NSSI.

## Notes

Supplement. **eTable 1.** The Young’s Internet Addiction Test **eTable 2.** The Resilience Scale for Chinese Adolescents **eTable 3.** Emotional Management Abilities **eTable 4.** Loneliness Scale **eTable 5.** Number, % of Less Frequent and More Frequent NSSI Among Males and Females **eTable 6.** Associations of Possible Internet Addiction and Internet Addiction With Emotional Regulation Ability and Loneliness Among Chinese Adolescents
