# Supplementary material for: Association of Internet Addiction With Nonsuicidal Self-injury Among Adolescents in China
Source: JAMA Netw Open. 2020 Jun 4;3(6):e206863. doi: 10.1001/jamanetworkopen.2020.6863 (PMC7273191; doi:10.1001/jamanetworkopen.2020.6863)
Supplement: Supplement. — eTable 1. The Young’s Internet Addiction Test eTable 2. The Resilience Scale for Chinese Adolescents eTable 3. Emotional Management Abilities eTable 4. Loneliness Scale eTable 5. Number, % of Less Frequent and More Frequent NSSI Among Males and Females eTable 6. Associations of Possible Internet Addiction and Internet Addiction With Emotional Regulation Ability and Loneliness Among Chinese Adolescents [file jamanetwopen-3-e206863-s001.pdf]

Supplementary Online Content

Tang J, Ma Y, Lewis SP et al. Association of internet addiction with nonsuicidal self-injury among adolescents in China. *JAMA Netw Open*. 2020;3(6):e206863. doi:10.1001/jamanetworkopen.2020.6863

eTable 1. The Young's Internet Addiction Test

eTable 2. The Resilience Scale for Chinese Adolescents

eTable 3. Emotional Management Abilities

eTable 4. Loneliness Scale

eTable 5. Number, % of less Frequent and More Frequent NSSI among Males and Females

eTable 6. Associations of Possible Internet Addiction and Internet Addiction with Emotional Regulation Ability and Loneliness among Chinese Adolescents

This supplementary material has been provided by the authors to give readers additional information about their work.

© 2020 Tang J et al. *JAMA Network Open.*

eTable 1. The Young's Internet Addiction Test

Using this scale of 5 choices, answer how often the situations below apply to you and your use of computers or smartphones:

Items	Never	Rarely	Sometimes	Often	Always
1. I find myself using my smartphone or computer longer than I planned to.	1	2	3	4	5
2. I would rather spend time online than do things around the house.	1	2	3	4	5
3. I would rather be on my smartphone or computer than interact with my partner.	1	2	3	4	5
4. I often make new friends online	1	2	3	4	5
5. People close to me are concerned about the amount of time I spend on my smartphone or computer.	1	2	3	4	5
6. My performance in school suffers because of the amount of time I spend online.	1	2	3	4	5
7. I will check email or messages online regardless of some issues around me.	1	2	3	4	5
8. My daily life was affected because of online.	1	2	3	4	5
9. I worry about online privacy being known by others.	1	2	3	4	5
10. I use the internet when I feel bad.	1	2	3	4	5
11. I will long for the next time after I used the internet.	1	2	3	4	5
12. I would be less interesting and happy without access to the internet.	1	2	3	4	5
13. I am bothered when people interrupt me while I am using my computer or smartphone.	1	2	3	4	5
14. I stay up later at night than I had intended due to doing things online.	1	2	3	4	5
15. When I am unable to use the internet, I miss it or think about what I could be doing online.	1	2	3	4	5
16. When I use the internet, I say to myself: "just play for a while".	1	2	3	4	5
17. I have tried to reduce my smartphone or internet use without success.	1	2	3	4	5
18. I did not tell others how many times I stay online every day.	1	2	3	4	5
19. I would rather stay home and use the internet than go out with friends.	1	2	3	4	5
20. I will be upset and anxious without access to the internet, until I can access to the internet.	1	2	3	4	5

© 2020 Tang J et al. *JAMA Network Open.*

eTable 2. The Resilience Scale for Chinese Adolescents

Circle "O" on the option number that best suits your situation.

**Items**	**Totally inconsistent**	**inconsistent**	**Not sure**	**consistent**	**Fully consistent**
1. Failure always makes me discourage.	1	2	3	4	5
2. I have difficulty controlling my unpleasant emotions.	1	2	3	4	5
3. I have clear goals on my life.	1	2	3	4	5
4. Generally, I become more mature and experienced after frustration.	1	2	3	4	5
5. Failure and frustration will make me doubt my abilities.	1	2	3	4	5
6. When I encounter something unpleasant, I can't find the right person to discuss.	1	2	3	4	5
7. I have a friend with the similar age and I can tell my difficulties to him/her.	1	2	3	4	5
8. My parents respect my ideas.	1	2	3	4	5
9. I don't know who can help me when I have difficulties.	1	2	3	4	5
10. I think the process is more helpful for people to grow than the results.	1	2	3	4	5
11. I usually make a plan and solution when I am facing difficulties	1	2	3	4	5
12. I used to keeping things in my mind instead of telling to others.	1	2	3	4	5
13. I think adversity can inspire people.	1	2	3	4	5
14. Sometimes, adversity helps to growth.	1	2	3	4	5
15. Parents always like to interfere with my thoughts.	1	2	3	4	5
16. At home, what I say is never heard.	1	2	3	4	5
17. I need confidence and spiritual support from my parents.	1	2	3	4	5
18. I will tell others initially when I am in troubles.	1	2	3	4	5
19. My Parents never blame me.	1	2	3	4	5
20. When faced with difficulties, I will concentrate.	1	2	3	4	5
21. It usually takes long time for me to forget the unpleasant things.	1	2	3	4	5
22. My parents always encourage me to go all out.	1	2	3	4	5
23. I can adjust my emotions very well in a short time.	1	2	3	4	5
24. I usually set goals to motivate myself.	1	2	3	4	5
25. I think everything has its positive side.	1	2	3	4	5
26. When I am in bad mood, I am unwilling to tell others.	1	2	3	4	5
27. My mood fluctuates too much	1	2	3	4	5

© 2020 Tang J et al. *JAMA Network Open.*

eTable 3. Emotional management abilities

Circle “O” on the option number that best suits your situation.

Items	Always like this	Often like this	Rarely like this	Never like this
1. I don’t know how to relieve when I feel stressed.	1	2	3	4
2. I can’t control my emotions when I feel angry.	1	2	3	4
3. I don’t know what to do to make me feel better when I am sad.	1	2	3	4
4. My bad mood will last for a long time before I can clam down.	1	2	3	4

© 2020 Tang J et al. *JAMA Network Open.*

eTable 4. Loneliness scale

Circle "O" on the option number that best suits your situation.

**Items**	**Totally inconsistent**	**inconsistent**	**Not sure**	**consistent**	**Fully consistent**
1. I make friends easily at school.	1	2	3	4	5
2. I have no one to talk with at school.	1	2	3	4	5
3. I cooperate with classmates very well.	1	2	3	4	5
4. I have many friends at school.	1	2	3	4	5
5. It is difficult for me to make other classmates like me.	1	2	3	4	5
6. No classmates play with me at school.	1	2	3	4	5
7. I always like to stay with classmates.	1	2	3	4	5
8. I often feel refusal from my classmates at school.	1	2	3	4	5
9. When I need help, I can't find anyone at school who is willing to help me.	1	2	3	4	5
10. When I was upset or anxious, no classmate who I can spoke to.	1	2	3	4	5
11. I get on well with my classmates at school.	1	2	3	4	5
12. I always go alone at school.	1	2	3	4	5
13. Nobody in school really understands me.	1	2	3	4	5
14. I don't have a real friend at school.	1	2	3	4	5
15. I often feel alone at school.	1	2	3	4	5
16. Although there are many students around, no one care about me.	1	2	3	4	5
17. All classmates like me.	1	2	3	4	5
18. I feel no one can be trusted at school.	1	2	3	4	5
19. I often fell left out at school.	1	2	3	4	5
20. I often feel that my interests and ideas are different from my classmates.	1	2	3	4	5
21. I often feel I have nothing to do at school.	1	2	3	4	5

© 2020 Tang J et al. *JAMA Network Open.*

eTable 5. Number, % of 'less frequent' and 'more frequent' NSSI among males and females

	Male (n=8043)	Female (n=7580)	All	χ^2	*p*
**Non-NSSI**	5846(72.4)	5312(70.1)	11158(71.4)		
**'Less frequent' NSSI**	1288(16.0)	1379(18.2)	2667(17.1)	14.481	<0.001
**'More frequent' NSSI**	909(11.3)	889(11.7)	1798(11.5)	2.093	0.148

Abbreviations: NSSI=non-suicidal self-injury

© 2020 Tang J et al. *JAMA Network Open.*

eTable 6. Associations of Possible Internet Addiction and Internet Addiction with Emotional Regulation Ability and Loneliness among Chinese Adolescents

	**Non-NSSI (n=11158)**	**'Less frequent' NSSI (n=2667)**	**'More frequent' NSSI (n=1798)**	**All**	***F***	***p***
**Emotional regulation ability, mean (SD)**	11.9±2.7	10.9±2.6	10.2±3.0	11.5±2.8	360.45	<0.001
**Loneliness, mean (SD)**	48.7±9.8	52.6±10.1	54.6±11.0	49.8±10.2	311.86	<0.001

© 2020 Tang J et al. *JAMA Network Open.*
